# Cancer risk among patients with type 2 diabetes mellitus: a population-based prospective study in China

**DOI:** 10.1038/srep11503

**Published:** 2015-06-17

**Authors:** Meng Wang, Ru-Ying Hu, Hai-Bin Wu, Jin Pan, Wei-Wei Gong, Li-Hua Guo, Jie-Ming Zhong, Fang-Rong Fei, Min Yu

**Affiliations:** 1 Zhejiang Provincial Center for Disease Control and Prevention, 3399 Binsheng Road, Hangzhou 310051, China

## Abstract

Evidence indicates an increased cancer risk among type 2 diabetes mellitus (T2DM) patients, yet studies in mainland China are scarce. Based on Diabetes Surveillance System linking to Cancer Surveillance System of Zhejiang Province in China, we explored the cancer risk among T2DM patients. Totally, 327,268 T2DM patients were identified and followed from January 1, 2007 to December 31, 2013. Standardized incidence ratios (SIRs) with 95% confidence intervals (CIs) were reported. Overall cancer risk was found significantly increased with an SIR of 1.15 (95% CI 1.12–1.19) and 1.25 (95% CI 1.21–1.30) in males and females, respectively. Regarding specific cancer sites, risks of liver, colon, rectum, pancreas, and kidney were significantly increased with SIRs of 1.26 (95% CI 1.16–1.36), 1.47 (95% CI 1.29–1.67), 1.25 (95% CI 1.09–1.43), 2.81 (95% CI 2.50–3.16) and 1.61 (95% CI 1.28–2.03) in males, 1.53 (95% CI 1.35–1.73), 1.33 (95% CI 1.15–1.54), 1.29 (95% CI 1.10–1.51), 3.62 (95% CI 3.20–4.09) and 1.71 (95% CI 1.28–2.29) in females, respectively. A significant increased SIR was noted for prostate (1.80, 95% CI 1.58–2.06). Significant increased SIRs for lung (1.32, 95% CI 1.20–1.44) and stomach (1.16, 95% CI 1.03–1.30) were observed in females. We suggested an increased cancer risk among T2DM patients.

The International Diabetes Federation has stated that 387 million people throughout the world have diabetes mellitus in 2014, which will rise to 592 million in 2035^1^. Type 2 diabetes mellitus (T2DM) results from the body’s ineffective use of insulin and comprises 90% of people with diabetes around the world^2^. Cancer is also a serious public health problem. The World Health Organization reported that there were 14.1 million new cases and 8.2 million deaths in 2012 globally^3^. Interestingly, there is now considerable professional evidence indicating that T2DM is associated with an increased risk of several cancer types, such as liver, pancreatic, colon, breast, and bladder cancers^4^,^5^. Moreover, many possible biochemical mechanisms also provide biological plausibility for a causal association between T2DM and cancer^6^,^7^,^8^. While the association between T2DM and cancer has been extensively studied and the epidemiological evidence was likely to be solid, however, the association may be biased by a series of confounders (e.g. detection bias). Furthermore, due to studies with small sample sizes, there was insufficient statistical power to examine the true association between T2DM and cancer.

The above evidence, however, was mainly based on findings from western world and Taiwan. To the best of our knowledge, studies on the association between T2DM and cancer in Chinese population from mainland China are still lacking. Thus, the primary objective of this study is to explore the cancer risk among Chinese patients with T2DM in a population-based prospective study.

## Methods

### Data Sources

This was a population-based, prospective study conducted by using Diabetes and Cancer Surveillance System of Zhejiang Province in China, which were established in 2001with thirty representative surveillance districts and over 16 million residents^9^. Once the specific diabetes and cancer patients were diagnosed by a certificated health practitioner, within a week, the patient’s detailed information including demographics, diagnosis, and laboratory indicators was registered in the corresponding surveillance system. After verified by regional Center for Disease control and Prevention (CDC), the data were reported to provincial CDC for further verification to make sure that only the newly-diagnosed cases were recorded in the computerized databases with a unique identification number. The confirmed and recorded patients would be followed-up once per year. Furthermore, with the Diabetes Surveillance System, we included all the patients with type 1, type 2, gestational or other types of diabetes. The classification of diabetes type and registration was completed by a health practitioner. All the recorded diabetes and cancer patients were coded according to the International Classification of Disease 10^th^ revision (ICD-10). This study was carried out in accordance with the “Declaration of Helsinki”.

### Data Linkage

In the present study, only the T2DM and cancer patients recorded between January 1, 2007 and December 31, 2013 were included. Before linking the two databases, we excluded the possible duplicated records using the unique identification number and individuals lacking the complete information were also removed from the study. Then cancer database was linked to T2DM database using the patients’ identity card number, sex, the full name, date of birth (year and month) and code of district registered in the system. Given the aim of evaluating cancer risk among patients with T2DM, we also excluded those paired records with the date of initial T2DM diagnosis later than cancer.

### Statistical Analysis

The calculation of person-years of the individuals was conducted in both paired and unpaired groups, respectively. For the first condition, person-years were calculated from the date of initial diagnosis of T2DM to the occurrence of specific cancer cases. For the second condition, person-years were calculated from the date of initial diagnosis of T2DM to the closing date of this study (December 31, 2013). In addition, no matter paired or unpaired group, person-years were also calculated from the date of initial diagnosis of T2DM to the dates of death and emigration, if death and emigration came first. Cancer risk among T2DM patients was estimated by comparing with the general population in the surveillance districts using standardized incidence ratios (SIRs) and 95% confidence intervals (CIs), adjusted by sex, age and urbanization level of area registered in the system. SIRs and 95% CIs were calculated as number of observed cases divided by number of expected cases with the Poisson regression model. In particular, since there was no cancer case with T2DM in the age group of less than 20 years, only three age groups (20–40, 40–60, >60 years) were included in our analysis. Furthermore, to investigate the possible effects of diabetes duration on the cancer risk in T2DM patients, the separate SIRs for different follow-up periods (≤5, 5–7 years) were also calculated. All analyses were performed using SAS statistical package (version 9.2, SAS Institute, Inc., Cary, NC, USA).

## Results

This study consisted of 327,268 T2DM and 7,435 cancer patients with T2DM between January 1, 2007 and December 31, 2013. The mean ages at diagnosis and registration of diabetes were 59.40 ± 13.26 years and 59.82 ± 13.28 years, respectively. The detailed baseline characteristics of T2DM and cancer patients were described in Table 1.

Table 2 showed the total and sex-specific cancer risks among patients with T2DM. For all patients, the SIR (95% CI) for total cancer was 1.19 (1.17–1.22). Among 21 cancer sites, 11 showed significant increased SIRs for patients with T2DM. The highest SIR was for cancer site of pancreas (3.14, 95% CI 2.89–3.42). Three cancer sites with significant decreased SIRs were noted for stomach (0.92, 95% CI 0.85–0.98), esophagus (0.76, 95% CI 0.66–0.87), and cervix (0.68, 95% CI 0.57–0.82) respectively. For males, the SIR for total cancer was 1.15 (95% CI 1.12–1.19). Cancer sites with significant increased SIRs included liver, colon, rectum, small intestine, pancreas, thyroid, prostate, bladder, and kidney. Cancer sites with significant decreased SIRs were stomach and esophagus. For females, the SIR for total cancer was 1.25 (95% CI 1.21–1.30). Cancer sites with significant increased SIRs included lung, liver, gallbladder, stomach, colon, rectum, pancreas, kidney, and endometrium. Cancer site with significant decreased SIR was seen in cervix.

Table 3 showed the cancer risks among urban and rural areas patients with T2DM. For urban area, the SIR for total cancer was 1.16 (95% CI 1.12–1.20). Cancer sites with significant increased SIRs included liver, gallbladder, colon, rectum, pancreas, prostate, bladder, kidney, and endometrium. Significant decreased SIRs were observed in cancer sites of breast and cervix. For rural area, the SIR for total cancer was 1.20 (95% CI 1.17–1.24). Cancer sites with significant increased SIRs included lung, liver, gallbladder, stomach, colon, rectum, esophagus, pancreas, breast, prostate, kidney, and endometrium. No significant decreased SIR was seen in any site-specific cancer.

Table 4 showed the cancer risks in different follow-up intervals among patients with T2DM. Compared to the short follow-up interval, lower but remained significant SIRs were observed in the relatively long follow-up interval in total cancer and 18 site-specific cancers, except for the sites of cervix, skin, and nasopharynx.

Table 5 showed the age-specific cancer risks among patients with T2DM. For cancer sites of liver, stomach, colon, esophagus, pancreas, breast, and thyroid, the SIRs of cancer decreased with age.

## Discussion

Based on 327,268 T2DM patients, this study was performed to explore the subsequent risk of cancer in population from mainland China. Totally, increased risk of cancer and of most site-specific cancers were observed, with SIRs ranging from 1.09 to 3.14. When stratified by sex, age and urbanization, the excess risk of cancer remained and decreased with age. Meantime, stratified by diabetes duration, a much higher cancer risk in the first five years after diabetes diagnosis was also observed. Given the rapid growth of diabetes in China, even a small increased cancer risk would have important public health implications at population level.

Several studies on the association between T2DM and cancer using SIRs have indicated an increased risk of cancer at various sites, including lung^10^,^11^,^12^, liver^10^,^11^,^12^, pancreas^10^,^11^,^12^, ovary^11^,^12^, breast^10^,^11^,^12^, stomach^11^,^12^, rectum^11^,^12^, colon^11^,^12^, small intestine^11^,^12^, bladder^10^,^11^,^12^, kidney^10^,^11^,^12^, and endometrium^10^,^11^,^12^, whereas a decreased risk at site of prostate^10^,^11^. This study covered all the types of cancer and findings were comparable to those in large prospective studies except for prostate cancer. Different from previous studies, we observed a significant positive association between T2DM and prostate cancer. This discrepancy may be attributable to our small sample size and relatively short follow-up time. In the current study, pancreatic cancer showed the strongest risk in T2DM patients, comparable to a population-based cohort study of 380,196 Swedes^11^. As biological evidence, a dose-response meta-analysis revealed that every 0.56 mmol/L increase in fasting blood glucose was associated with a 14% increase in the rate of pancreatic cancer^13^. Although the association between T2DM and lung cancer has been suggested, the conclusions were inconsistent in studies^14^,^15^,^16^,^17^,^18^. Overall, our findings supported the hypothesis that the risk of developing lung cancer increase in T2DM patients, particularly among women. Our study also suggested an increased risk of breast cancer in postmenopausal women with T2DM in Chinese population, though was not statistical significant. This was consistent with the results from cohort studies conducted in British Columbia and China^19^,^20^. A meta-analysis also showed an increased risk of breast cancer in women with T2DM, even after adjustment for body mass index^21^. For stomach cancer, the association with diabetes was uncertain in western population. Among Asian population, except for Japanese population with a high incidence of this malignancy^22^, a modest increased risk of stomach cancer was observed in a study involving 895,434 T2DM patients in Taiwan^15^. Our analysis only showed a significant increased cancer risk in females, comparable to a recent meta-analysis of seventeen cohort studies and four case-control studies^23^ and a more recent meta-analysis of eleven cohort studies and six case-control studies^24^. Esophageal cancer showed a decreased risk in T2DM patients, which were consistent with findings from Asian population^15^,^25^ and Australian females^10^. The potential link between T2DM and ovarian cancer was inconclusive due to scarce investigation in the past. Overall, we observed a null association between T2DM and ovarian cancer, which was consistent with results from a Taiwan cohort study^26^. Differently, stratified by diabetes duration, the significant increased risk was observed in our study, but not in Taiwan study. Finally, we also exhibited consistent null associations between non-Hodgkin’s lymphoma, brain cancers and T2DM in Asians^15^. Age stratification analysis in our study revealed that the risk of certain cancers decreased with age. Compared to non-DM cohorts, a study in Taiwan using another indicator of incidence rate ratio (IRR) also presented that risk of cancer among DM cohorts decreased with age^15^.

The increasing studies have reported that the cancer risk will vary by duration of diabetes and proposed to examine the temporal association between diabetes and cancer risk^27^,^28^,^29^. In the present study, we observed a much higher increased cancer risk in the first five years after diabetes diagnosis. According to the earlier literature, this could be partially explained by detection bias due to increased medical attention (cancer screening or hospitalizations) around the time of recognition of diabetes^10^,^17^. Above all, the SIRs remained the significant increasing after five years for total cancer and most site-specific cancers in this study, which showed that the positive association was unlikely to be due to reverse causality, particularly for pancreatic cancer.

Notably, it was unavailable to distinguish T2DM with latent autoimmune diabetes in adults (LADA) and the bias of exposure misclassification is inevitable in our study. However, according to the literature, the LADA occurs in 10% of individuals older than 35 years and in 25% below that age^30^. In the present study, the diabetes patients diagnosed at ages above 40 years accounted for 93.32%, indicating that approximate 90% of the diabetes patients only had T2DM in this study. The results suggest that cancer risk among T2DM patients is similar with or without LADA.

Possible biological mechanisms involved in the diabetes-cancer link have been extensively studied and were hypothesized to rely on hyperglycemia, hyperinsulinemia and inflammation^6^,^7^,^8^. Furthermore, other factors such as diabetes treatments^31^,^32^ and physical inactivity^33^ may also play a contributory role in linking T2DM and cancer.

The present study had several strengths. This study was one of the few studies exploring the cancer risk in T2DM patients with SIR in mainland China. It was a population-based prospective study with large sample of 327,268 T2DM cases. T2DM and cancer cases were diagnosed by certificated health practitioners, and related data was verified by regional, provincial CDCs, and recorded in the corresponding surveillance system eventually.

However, some limitations were also observed. Firstly, some site-specific cancers number was relatively small, which would decrease the statistical power to examine the true association between T2DM and cancer. Secondly, we only adjusted for variables of sex, age, urbanization and diabetes duration, while other potential confounding factors including obesity status, smoking, alcohol consumption, physical activity and diabetes treatments have not been considered in the analysis. Thirdly, the follow-up time was relative short and only lasted seven years, which restricted our ability to further assess the potential lead time bias.

In summary, the current study indicated that T2DM increased the risk of developing cancer in Chinese population from mainland China. Although the association between T2DM and cancer may be biased by a series of confounders, our findings still have important implication that early and careful screening for cancer in T2DM patients is necessary in clinical practice.

## Additional Information

**How to cite this article**: Wang, M. *et al.* Cancer risk among patients with type 2 diabetes mellitus: a population-based prospective study in China. *Sci. Rep.* **5**, 11503; doi: 10.1038/srep11503 (2015).

## Figures and Tables

**Table 1:** Characteristics of type 2 diabetes and cancer cases included in study.

	**Type 2 diabetes**
Number of cases (%)	327,268 (94.75)
Mean age at diagnosis (SD), year	59.40 (13.26)
Mean age at registration (SD), year	59.82 (13.28)
Males (%)	163,819 (50.06)
Females (%)	163,449 (49.94)
Urban area (%)	130,807(39.97)
Rural area (%)	196,461(60.03)
Number of cancer cases	7435
Males (%)	4,106 (55.23)
Females (%)	3,329 (44.77)
Urban area (%)	3,187 (42.86)
Rural area (%)	4,248 (57.14)

**Table 2:** SIRs for male and female patients with type 2 diabetes from 2007 to 2013.

**Site of cancer**	**Total**			**Males**			**Females**		
	**N**	**SIR**	**95% CI**	**N**	**SIR**	**95% CI**	**N**	**SIR**	**95% CI**
All cancer	7,435	**1.19**	**1.17–1.22**	4,106	**1.15**	**1.12–1.19**	3,329	**1.25**	**1.21–1.30**
Lung	1,326	**1.09**	**1.03–1.15**	858	1.00	0.94–1.08	468	**1.32**	**1.20–1.44**
Liver	861	**1.32**	**1.23–1.41**	615	**1.26**	**1.16–1.36**	246	**1.53**	**1.35–1.73**
Gallbladder	87	**1.71**	**1.38–2.11**	23	1.39	0.92–2.09	64	**1.84**	**1.44–2.36**
Stomach	729	**0.92**	**0.85–0.98**	451	**0.82**	**0.75–0.90**	278	**1.16**	**1.03–1.30**
Colon	413	**1.40**	**1.27–1.55**	236	**1.47**	**1.29–1.67**	177	**1.33**	**1.15–1.54**
Rectum	366	**1.26**	**1.14–1.40**	215	**1.25**	**1.09–1.43**	151	**1.29**	**1.10–1.51**
Esophagus	209	**0.76**	**0.66–0.87**	155	**0.70**	**0.60–0.82**	54	0.99	0.76–1.30
Small intestine	47	**1.42**	**1.07–1.89**	29	**1.57**	**1.09–2.27**	18	1.24	0.78–1.96
Pancreas	540	**3.14**	**2.89–3.42**	283	**2.81**	**2.50–3.16**	257	**3.62**	**3.20–4.09**
Breast	396	1.01	0.92–1.12	0			396	1.01	0.92–1.12
Thyroid	301	1.04	0.93–1.13	97	**1.49**	**1.22–1.81**	204	0.90	0.79–1.03
Prostate	213	**1.78**	**1.55–2.03**	213	**1.80**	**1.58–2.06**	0		
Bladder	158	**1.23**	**1.06–1.44**	124	**1.23**	**1.03–1.47**	34	1.29	0.92–1.81
Kidney	118	**1.64**	**1.37–1.97**	73	**1.61**	**1.28–2.03**	45	**1.71**	**1.28–2.29**
Ovary	71	1.07	0.85–1.36	0			71	1.06	0.84–1.34
Cervix	115	**0.68**	**0.57–0.82**	0			115	**0.67**	**0.56–0.81**
Endometrium	95	**1.68**	**1.38–2.06**	0			95	**1.66**	**1.36–2.03**
Skin	60	1.06	0.82–1.36	30	1.02	0.72–1.46	30	1.10	0.77–1.57
Non-Hodgkin’s lymphoma	61	1.10	0.85–1.41	41	1.20	0.88–1.62	20	0.95	0.61–1.47
Nasopharynx	84	0.93	0.75–1.15	59	0.93	0.72–1.19	25	0.95	0.64–1.41
Brain	110	1.13	0.93–1.36	55	1.05	0.81–1.37	55	1.21	0.93–1.58

Bold type indicates that the CI does not include the null.

Abbreviations: N, observed number of cases; SIR, standardized incidence ratio; CI, confidence interval.

**Table 3:** SIRs for urban and rural area patients with type 2 diabetes from 2007 to 2013.

**Site of cancer**	**Total**			**Urban Area**			**Rural Area**		
	**N**	**SIR**	**95% CI**	**N**	**SIR**	**95% CI**	**N**	**SIR**	**95% CI**
All cancer	7,435	**1.19**	**1.17–1.22**	3,187	**1.16**	**1.12–1.20**	4,248	**1.20**	**1.17–1.24**
Lung	1,326	**1.09**	**1.03–1.15**	533	1.04	0.95–1.13	793	**1.94**	**1.81–2.07**
Liver	861	**1.32**	**1.23–1.41**	310	**1.24**	**1.11–1.38**	551	**2.76**	**2.54–3.00**
Gallbladder	87	**1.71**	**1.38–2.11**	46	**1.75**	**1.31–2.34**	41	**1.58**	**1.16–2.15**
Stomach	729	**0.92**	**0.85–0.98**	305	1.05	0.94–1.08	424	**1.83**	**1.67–2.02**
Colon	413	**1.40**	**1.27–1.55**	202	**1.35**	**1.18–1.55**	211	**1.78**	**1.55–2.03**
Rectum	366	**1.26**	**1.14–1.40**	159	**1.23**	**1.05–1.44**	207	**2.01**	**1.75–2.30**
Esophagus	209	**0.76**	**0.66–0.87**	77	0.90	0.72–1.13	132	**1.94**	**1.64–2.31**
Small intestine	47	**1.42**	**1.07–1.89**	23	1.47	0.98–2.22	24	1.34	0.90–2.00
Pancreas	540	**3.14**	**2.89–3.42**	218	**2.54**	**2.23–2.90**	322	**4.71**	**4.23–5.26**
Breast	396	1.01	0.92–1.12	171	**0.84**	**0.72–0.97**	225	**1.38**	**1.21–1.58**
Thyroid	301	1.04	0.93–1.13	149	0.87	0.74–1.03	152	1.12	0.95–1.31
Prostate	213	**1.78**	**1.55–2.03**	121	**1.84**	**1.54–2.20**	92	**1.76**	**1.43–2.16**
Bladder	158	**1.23**	**1.06–1.44**	79	**1.27**	**1.02–1.58**	79	1.16	0.93–1.45
Kidney	118	**1.64**	**1.37–1.97**	63	**1.53**	**1.19–1.96**	55	**1.66**	**1.27–2.16**
Ovary	71	1.07	0.85–1.36	33	0.97	0.69–1.36	38	1.13	0.82–1.56
Cervix	115	**0.68**	**0.57–0.82**	30	**0.48**	**0.33–0.68**	85	0.81	0.65–1.00
Endometrium	95	**1.68**	**1.38–2.06**	37	**1.49**	**1.08–2.06**	58	**1.81**	**1.40–2.34**
Skin	60	1.06	0.82–1.36	26	1.23	0.84–1.80	34	0.97	0.69–1.35
Non-Hodgkin’s lymphoma	61	1.10	0.85–1.41	36	1.30	0.94–1.80	25	0.86	0.58–1.28
Nasopharynx	84	0.93	0.75–1.15	34	0.87	0.62–1.21	50	0.96	0.73–1.27
Brain	110	1.13	0.93–1.36	50	1.16	0.88–1.53	60	1.08	0.84–1.40

Bold type indicates that the CI does not include the null.

Abbreviations: N, observed number of cases; SIR, standardized incidence ratio; CI, confidence interval.

**Table 4:** SIRs for different follow–up intervals in type 2 diabetes patients from 2007 to 2013.

**Site of cancer**	**Total**			**≤5 (years)**			**5–7 (years)**		
	**N**	**SIR**	**95% CI**	**N**	**SIR**	**95% CI**	**N**	**SIR**	**95% CI**
All cancer	7,435	**1.19**	**1.17–1.22**	5,450	**3.19**	**3.11–3.28**	1,985	**2.08**	**1.99–2.17**
Lung	1,326	**1.09**	**1.03–1.15**	974	**2.93**	**2.75–3.12**	352	**1.89**	**1.70–2.10**
Liver	861	**1.32**	**1.23–1.41**	598	**3.34**	**3.08–3.62**	263	**2.62**	**2.32–2.96**
Gallbladder	87	**1.71**	**1.38–2.11**	64	**4.59**	**3.59–5.86**	23	**2.94**	**1.96–4.43**
Stomach	729	**0.92**	**0.85–0.98**	524	**2.40**	**2.21–2.62**	205	**1.68**	**1.47–1.93**
Colon	413	**1.40**	**1.27–1.55**	292	**3.63**	**3.23–4.07**	121	**2.68**	**2.25–3.21**
Rectum	366	**1.26**	**1.14–1.40**	284	**3.58**	**3.18–4.02**	82	**1.84**	**1.49–2.29**
Esophagus	209	**0.76**	**0.66–0.87**	140	**1.85**	**1.57–2.18**	69	**1.63**	**1.29–2.06**
Small intestine	47	**1.42**	**1.07–1.89**	32	**3.54**	**2.50–5.01**	15	**2.96**	**1.79–4.92**
Pancreas	540	**3.14**	**2.89–3.42**	420	**8.92**	**8.11–9.82**	120	**4.55**	**3.81–5.45**
Breast	396	1.01	0.92–1.12	289	**2.70**	**2.41–3.03**	107	**1.79**	**1.48–2.16**
Thyroid	301	1.04	0.93–1.13	243	**3.07**	**2.71–3.48**	58	**1.31**	**1.01–1.69**
Prostate	213	**1.78**	**1.55–2.03**	158	**4.81**	**4.12–5.63**	55	**2.99**	**2.30–3.90**
Bladder	158	**1.23**	**1.06–1.44**	115	**3.28**	**2.73–3.94**	43	**2.19**	**1.62–2.95**
Kidney	118	**1.64**	**1.37–1.97**	90	**4.58**	**3.72–5.63**	28	**2.54**	**1.76–3.68**
Ovary	71	1.07	0.85–1.36	47	**2.60**	**1.95–3.46**	24	**2.37**	**1.59–3.53**
Cervix	115	**0.68**	**0.57–0.82**	91	**1.97**	**1.60–2.42**	24	0.93	0.62–1.38
Endometrium	95	**1.68**	**1.38–2.06**	70	**4.53**	**3.59–5.73**	25	**2.89**	**1.95–4.28**
Skin	60	1.06	0.82–1.36	50	**3.22**	**2.44–4.25**	10	1.15	0.62–2.14
Non-Hodgkin’s lymphoma	61	1.10	0.85–1.41	43	**2.83**	**2.10–3.81**	18	**2.11**	**1.33–3.36**
Nasopharynx	84	0.93	0.75–1.15	65	**2.62**	**2.05–3.34**	19	1.37	0.87–2.15
Brain	110	1.13	0.93–1.36	75	**2.80**	**2.24–3.52**	35	**2.34**	**1.68–3.26**

Bold type indicates that the CI does not include the null.

Abbreviations: N, observed number of cases; SIR, standardized incidence ratio; CI, confidence interval.

**Table 5:** SIRs for different ages of type 2 diabetes patients from 2007 to 2013.

**Site of cancer**	**Total**			**20–40 (years)**			**40–60 (years)**			**>60 (years)**		
	**N**	**SIR**	**95% CI**	**N**	**SIR**	**95% CI**	**N**	**SIR**	**95% CI**	**N**	**SIR**	**95%CI**
All cancer	7,435	**1.19**	**1.17–1.22**	93	**6.02**	**4.91–7.37**	2165	**1.73**	**1.66–1.81**	5177	1.00	0.98–1.03
Lung	1,326	**1.09**	**1.03–1.15**	3	2.68	0.87–8.32	289	**1.61**	**1.44–1.81**	1034	**0.84**	**0.79–0.89**
Liver	861	**1.32**	**1.23–1.41**	7	**5.51**	**2.63–11.56**	291	**1.91**	**1.71–2.15**	563	**1.10**	**1.02–1.20**
Gallbladder	87	**1.71**	**1.38–2.11**	0			17	**2.87**	**1.79–4.62**	70	1.26	0.99–1.59
Stomach	729	**0.92**	**0.85–0.98**	4	**4.78**	**1.79–12.72**	162	**1.30**	**1.12–1.52**	563	**0.72**	**0.66–0.78**
Colon	413	**1.4**	**1.27–1.55**	3	**6.27**	**2.02–19.43**	85	**1.79**	**1.45–2.22**	325	**1.16**	**1.04–1.30**
Rectum	366	**1.26**	**1.14–1.40**	2	**4.96**	**1.24–19.82**	87	**1.76**	**1.42–2.17**	277	1.02	0.90–1.15
Esophagus	209	**0.76**	**0.66–0.87**	1	**17.35**	**2.44–123.15**	58	**1.36**	**1.05–1.76**	150	**0.54**	**0.46–0.63**
Small intestine	47	**1.42**	**1.07–1.89**	0			16	**2.66**	**1.63–4.34**	31	1.04	0.73–1.47
Pancreas	540	**3.14**	**2.89–3.42**	4	**36.93**	**13.86–98.41**	117	**5.23**	**4.37–6.27**	419	**2.31**	**2.10–2.55**
Breast	396	1.01	0.92–1.12	7	**4.02**	**1.92–8.44**	201	**1.39**	**1.21–1.60**	188	**1.24**	**1.07–1.43**
Thyroid	301	1.04	0.93–1.13	40	**12.18**	**8.94–16.61**	179	**1.79**	**1.55–2.07**	82	**1.26**	**1.02–1.57**
Prostate	213	**1.78**	**1.55–2.03**	0			8	**2.19**	**1.10–4.39**	205	**1.32**	**1.15–1.50**
Bladder	158	**1.23**	**1.06–1.44**	0			31	**1.82**	**1.28–2.59**	127	0.96	0.81–1.14
Kidney	118	**1.64**	**1.37–1.97**	1	5.72	0.81–40.64	38	**2.19**	**1.59–3.01**	79	**1.52**	**1.22–1.89**
Ovary	71	1.07	0.85–1.36	1	2.44	0.34–17.29	28	1.38	0.96–2.00	42	**1.37**	**1.01–1.85**
Cervix	115	**0.68**	**0.57–0.82**	5	**4.15**	**1.73–9.97**	68	1.10	0.86–1.39	42	0.80	0.59–1.08
Endometrium	95	**1.68**	**1.38–2.06**	0			57	**2.77**	**2.13–3.59**	38	**1.55**	**1.13–2.14**
Skin	60	1.06	0.82–1.36	0			12	1.61	0.92–2.84	48	0.85	0.64–1.12
Non-Hodgkin’s lymphoma	61	1.1	0.85–1.41	0			21	**1.82**	**1.19–2.80**	40	0.95	0.70–1.30
Nasopharynx	84	0.93	0.75–1.15	2	**5.33**	**1.33–21.29**	39	**1.43**	**1.04–1.96**	43	0.87	0.64–1.17
Brain	110	1.13	0.93–1.36	3	**5.79**	**1.87–17.96**	36	**1.72**	**1.24–2.38**	71	1.12	0.89–1.41

Bold type indicates that the CI does not include the null. Abbreviations: N, observed number of cases; SIR, standardized incidence ratio; CI, confidence interval.
